# Association between risk polymorphisms for neurodegenerative diseases and cognition in colombian patients with frontotemporal dementia

**DOI:** 10.3389/fneur.2022.675301

**Published:** 2022-08-22

**Authors:** Andrea López-Cáceres, Francy Cruz-Sanabria, Pilar Mayorga, Ana Isabel Sanchez, Silvia Gonzalez-Nieves, Paola Ayala-Ramírez, Ignacio Zarante, Diana Matallana

**Affiliations:** ^1^Faculty of Medicine, Institute of Human Genetics, Pontificia Universidad Javeriana, Bogotá, Colombia; ^2^Hospital Universitario Fundación Santa Fe de Bogotá, Bogotá, Colombia; ^3^Department of Translational Research and New Technologies in Medicine and Surgery, University of Pisa, Pisa, Italy; ^4^Neuroscience Group, Universidad Nacional de Colombia, Bogotá, Colombia; ^5^Mental Health Department, Hospital Universitario Fundación Santa Fe de Bogotá, Bogotá, Colombia; ^6^Faculty of Health Sciences, Pontificia Universidad Javeriana, Cali, Colombia; ^7^Imbanaco Medical Center, Cali, Colombia; ^8^Faculty of Sciences, Universidad de los Andes, Bogotá, Colombia; ^9^Department of Psychiatry, School of Medicine, Instituto de Envejecimiento, Pontificia Universidad Javeriana, Bogotá, Colombia

**Keywords:** frontotemporal dementia, neuropsychological tests, cognition, SNP array, neurodegenerative disease

## Abstract

Frontotemporal dementia (FTD) is a heterogeneous neurodegenerative disease of presenile onset. A better characterization of neurodegenerative disorders has been sought by using tools such as genome-wide association studies (GWAS), where associations between single nucleotide polymorphisms (SNPs) and cognitive profiles could constitute predictive biomarkers for these diseases. However, in FTD, associations between genotypes and cognitive phenotypes are yet to be explored. Here, we evaluate a possible relationship between genetic variants and some cognitive functions in an FTD population.

**Methodology:**

A total of 47 SNPs in genes associated with neurodegenerative diseases were evaluated using the Sequenom MassARRAY platform along with their possible relationship with performance in neuropsychological tests in 105 Colombian patients diagnosed with FTD.

**Results and discussion:**

The SNPs rs429358 (*APOE*), rs1768208 (*MOBP*), and rs1411478 (*STX6*), were identified as risk factors for having a low cognitive performance in inhibitory control and phonological verbal fluency. Although the significance level was not enough to reach the corrected alpha for multiple comparison correction, our exploratory data may constitute a starting point for future studies of these SNPs and their relationship with cognitive performance in patients with a probable diagnosis of FTD. Further studies with an expansion of the sample size and a long-term design could help to explore the predictive nature of the potential associations we identified.

## Introduction

Frontotemporal dementia (FTD) is an early-onset, heterogeneous neurodegenerative disorder with a strong genetic component (1). Positive family history has been reported in FTD in up to 40% of cases (2, 3), with the most frequent mutations found in the following genes: microtubule-associated protein tau (*MAPT)*, granulin (*GRN*), and *C9orf72* (2, 4). According to clinical involvement, FTD is classified into behavioral and language variants (semantic dementia, primary progressive aphasia) (5–7). It also coexists with motor neuron disease (FTD-MND) and atypical parkinsonian disorders (1, 8, 9).

The clinical and molecular heterogeneity of FTD, as well as the overlapping of symptoms with other neurodegenerative diseases (1, 2), have led to it being characterized through genome-wide association studies (GWAS) (10, 11). These typically involve the use of single nucleotide polymorphisms (SNPs) that are common in a given population and can establish risk by association with different phenotypes related to the onset, development, and progression of the disease (10, 12, 13). More than 40 risk loci have been identified for dementia within the genome (10), reporting the APOE ε4 allele with the strongest risk factor for late-onset alzheimer's disease (AD), and as a modulator of the expression of other degenerative dementias (14, 15). Specifically for FTD, three significant SNPs (rs6966915, rs1020004, and rs1990622) have been reported in the transmembrane protein 106B (*TMEM106B*) gene (7p21.3), a protein involved in endolysosomal transport and in the modulation of GRN protein levels (10, 16). Besides, some other loci such as 6p21.3, encompassing HLA locus, and 11q14 encompassing *RAB38/CTSC* were statistically significant in GWAS for FTD (10, 11).

An association between risk polymorphisms and cognitive profiles in mild cognitive impairment (MCI), AD, FTD, and amyotrophic lateral sclerosis (ALS) have recently been explored to evaluate disease development and progression (9, 17). The association between the studied loci and deficits in cognitive processes such as executive functions, language, visuospatial skills, and memory have been found in the four diseases (12). The association between polymorphic variants and cognitive performance suggests that exploring this may be a useful measure to detect risk variants that could eventually be considered predictive biomarkers for neurodegenerative diseases. It also makes it possible to evaluate disease development and progression (16, 18, 19). Thus, our study's main focus is to evaluate a relationship between cognitive performance and SNPs associated with neurodegenerative diseases in a sample of Colombian patients with a probable diagnosis of FTD.

## Materials and methods

An analytical, observational, non-probabilistic convenience study was conducted between January 2012 and December 2014 in 105 patients with a probable diagnosis of FTD, determined through consensus by a multidisciplinary group of specialists (Neurologist, Geriatrician, Psychiatrist, and Neuropsychologist) at the Memory Clinic at Hospital Universitario San Ignacio (Bogotá, Colombia). FTD patients were diagnosed according to established criteria in the behavioral variant of FTD (bv-FTD), non-fluent/agrammatic-variant primary progressive aphasia (nfvPPA), and semantic-variant primary progressive aphasia (svPPA) (6, 7), following the guidelines developed by an International Consortium for the Diagnosis of Frontotemporal Dementia (5, 7). Exclusion criteria include visual and hearing impairments, severe alteration of mobility, delirium, absence of caregiver or informant, significant cerebrovascular disease, and other previously recognized neurological diseases. This study was approved by the ethics committee at Hospital Universitario San Ignacio and Pontificia Universidad Javeriana. All participants received and signed informed consent.

### Neuropsychological study

A total of seven validated neuropsychological tests (see Table 1) on memory, praxis, verbal fluency, attention, and executive function were used to assess the cognitive profile of each patient (20–22, 28, 29). Taking into account as a reference the normative data for the tests of the Neuronorma Colombia neuropsychological evaluation battery (22, 29). The values obtained were converted to scale scores and subsequently dichotomized into 1 and 0. Performances that were lower than expected, considering age and education with respect to population parameters (percentile ≤ 6), were coded as 1, while performances above said percentile were coded as 0 (22, 29).

**Table 1:** Neuropsychological tests.

**Neuropsychological instruments**	**Cognitive domain assessed**
Symbol Digit Modalities Test (SDMT) (20, 21).	Divided attention, visual search, and perceptual speed
Stroop Color Test (22, 23)	Executive functions: inhibitory control and processing speed
Rey-Osterrieth Complex Figure (23, 24)	Visuospatial and constructional skills
INECO Frontal Screening (IFS) (25)	An executive screening test that investigates processes of thought regulation and control, motor programming, sensitivity to interference and inhibitory control, working memory, interpretation of metaphorical information and planning
Semantic verbal fluency test (fruits/animals) (23, 26)	Language task that studies active search for verbal information by categories
Phonological verbal fluency test (p/m) (23, 26).	Language/executive functions: Processes of active information search starting from phonological routes that require inhibitory control
Grober-Buschke test for short- and long-term explicit verbal memory (Free and Cued Selective Reminding Test) (21, 27).	Explicit verbal memory with controlled coding

### Molecular study

All evaluated patients had a 3-cc blood sample taken in EDTA tubes from which the genomic DNA was extracted using the Salting Out protocol. The DNA was then quantified using a NanoDrop^®^ ND-1000 spectrophotometer. SNP genotyping was performed using a custom-designed panel on the Sequenom MassARRAY platform, developed at the University of Pennsylvania, in which 47 SNP-type genetic variants were evaluated in genes associated with neurodegenerative diseases (16, 23, 24), FTD, Alzheimer's disease (AD), amyotrophic lateral sclerosis (ALS), Parkinson's disease, and progressive supranuclear palsy (PSP) (see Annex 1) (16, 23). The assay consisted of an initial locus-specific PCR reaction, followed by single-base extension using mass-modified dideoxynucleotide terminators of an oligonucleotide primer which anneals immediately upstream of the polymorphic site of interest (25). Although not all included SNPs are relevant for FTD, it is more cost and time-efficient to use a single panel that can be applied generally since there is a significant overlap in neurodegenerative disease phenotypes (13, 26, 27, 30).

### Statistical analysis

#### Population

The clinical and sociodemographic characteristics were analyzed by calculating frequencies and central tendency measures (median-range). ANOVA and chi-squared tests were used to determine group differences in sociodemographic variables.

#### Molecular

The allelic and genotypic frequencies were calculated by the counting method, and the Hardy-Weinberg (HW) equilibrium was determined for each SNP with the Arlequin v.3.5 software. The allelic frequencies obtained in the study were compared with the allelic frequencies reported in the 1,000 Genomes Global and in 1,000 Genomes Colombia samples by the χ^2^ association test, reporting their respective *p*-value (Stata/MP 14.0).

#### Molecular study and neuropsychological test

In order to identify a possible relationship between performance in cognitive tests and the SNPs assessed, a logistic regression model was calculated for each test and for each genetic variant in the R software. For all statistical tests, an alpha value of 0.05 was established. Based on these models, those SNPs that could significantly predict performance in each neuropsychological test were identified. To reduce Type I error for multiple comparisons, *p*-values were subjected to the Bonferroni correction with *n* = 47.

Similarly, the odds ratio (OR) of the allele related to these results was reported for each of the identified SNPs. Alleles with an OR < 1 were interpreted as being associated with adequate performance in the test, while ORs > 1 was associated with the risk of poor performance in neuropsychological tests.

## Results

Of the 105 patients with FTD, 61 patients met the criteria for bv-FTD, 28 met the criteria for PPA, and 16 patients met the criteria for SD. The median age of patients at the time of diagnosis was 61 years (range 40–86 years). No sex differences were found in the total sample or inside each clinical variant (see Table 2). As for the patient's education level, only 8.5% (9) had primary education, 19.2% (28) completed secondary education, and 35.6% (31) had undertaken university studies. It was not possible to determine the education level of 38 patients.

**Table 2:** Sociodemographic characteristics.

**Variable**	**nfvPPA (*N =* 28)**	**bvFTD (*N =* 61)**	**svPPA (*N =* 16)**	***p* value**
(*N =* 105)				
Sex (female), *N* (%)	16.00 (57.14)	30.00 (49.18)	9.00 (56.25)	0,74
Age (years), median (range)	62.00 (51.00–78.00)	65.00 (18.00–89.00)	60.00 (50.00–73.00)	0,52
Age of diagnosis (range)	60.50 (48–76)	61.50 (40–86)	59.50 (48–72)	0,60

nfvPPA, non-fluent/agrammatic-variant primary progressive aphasia (nfvPPA); bvFTD, behavioral variant FTD; svPPA, semantic-variant primary progressive aphasia.

We found four SNPs that were not in HW equilibrium: rs7412 in APOE (*p*-value = 0.029), rs6656401 in *CR1* (*p*-value = 0.024), rs983392 in *MS4A6A* (*p*-value = 0.009), and rs1411478 in *STX6* (*p*-value = 0.014). The first three SNPs are associated with AD and the last one with progressive supranuclear palsy (PSP). We also determined the frequency of the minor or risk allele in the SNPs associated with FTD and AD. By comparing them with the 1,000 Genomes Colombia and 1,000 Genomes Global samples, we reported a statistically significant difference in rs12546767 in *KIAA0196* gen (*p*-value < 0.001, *p*-value < 0.00001, respectively) (see Appendix 2).

Sixteen polymorphisms were significantly correlated with performance in one (or more than one) neuropsychological test (*p*-value < 0.05). Three of the alleles that were identified with a risk of poor performance in these tests correspond to the minor allele. A higher risk of poor performance in the phonological verbal fluency task was found for the *STX6* rs1411478 ^A^ allele. Similarly, *MOBP* rs1768208 ^T^ allele and *APOE* rs429358 ^C^ allele were identified as risk factors for poor performance in the Stroop Color Test (see Table 3). However, these findings did not survive the Bonferroni correction.

**Table 3:** OR values for SNPs with significant associations with neuropsychological test results.

**Test**	**SNP**	**Minor allele**	**OR (95% CI)**	***p*-value**	**Associated gene**
Symbol digit modalities test	rs1020004	G	0.29 (0.09–0.94)	0.039	TMEM106B
	rs2142991	C	0.11 (0.03–0. 46)	0.003	BMS1
	rs4938933	C	0.17 (0.05–0.57)	0.004	MS4A4A
Rey-osterrieth complex figure	rs2142991	C	0.24 (0.08–0.70)	0.009	BMS1
	rs4938933	C	0.28 (0.10–0,78)	0.016	MS4A4A
Verbal phonological fluency test	**rs1411478**	**A**	**4.78 (1.45**–**15.74)**	**0.010**	**STX6**
	rs7571971	T	0.32 (0.10–0.98)	0.046	EIF2AK3
Stroop color test	rs1468803	T	0.12 (0.02–0.58)	0.009	TMEM106B
	**rs1768208**	**T**	**5.8 (1.4**–**24.08)**	**0.015**	**MOBP**
	rs1990622	T	0.16 (0.03–0.72)	0.017	TMEM106B
	rs3807865	C	0.12 (0.02–0.58)	0.009	TMEM106B
	**rs429358**	**C**	**5.60 (1.21**–**25.94)**	**0.028**	**APOE**
	rs4663105	C	0.09 (0.01–0.62)	0.015	BIN1
	rs6852535	A	0.20 (0.05–0.82)	0.026	IL2, IL21
	rs7571971	T	0.22 (0.05–0.96)	0.044	EIF2AK3
INECO frontal screening total score	rs5848	A	0.08 (0.01–0.68)	0.021	GRN

The SNPs written in bold were associated with poor performance.

## Discussion

Frontotemporal dementia is a heterogeneous disease in both its clinical and genetic components (32–34). We find that the sociodemographic characteristics of this cohort of patients were consistent with what was reported in the literature. BvFTD was the most common clinical variant followed by language variants (35, 36). Regarding distribution by sex and the incidence of disease, there was no significant difference between the clinical groups (11, 26, 31). All the SNPs associated with FTD reached HW equilibrium in our population. The SNPs that did not reach HW equilibrium in our cohort of patients with a clinical diagnosis of FTD were located in genes associated with AD and PSP (*APOE, CR1, MS4A6A*, and *STX6* genes), which can be explained due to the sample size, or because these genes are subject to selection with each other between FTD and other neurodegenerative diseases (12, 37).

In addition, we compared the allele frequencies in our FTD sample with 1,000 Genomes and 1,000 Genomes Colombia populations, and we found that *KIAA0196* rs12546767 showed higher frequency in our sample, supporting the findings reported in previous studies in which this SNP has an increased disease association signal in the combined ALS and FTD (38).

Regarding correlations between cognitive performance and the SNP array panel, carriers of the *APOE* rs429358^C^ allele and *MOBP* rs1768208^T^ allele showed deficits in inhibitory control. Furthermore, carriers of *STX6* rs1411478^A^ allele performed poorly in phonological verbal fluency. Although the significant level of the identified risk between these alleles and the cognitive performance was not enough to reach the corrected alpha for multiple comparison correction, this information should not underrate because in studies with a larger sample with longitudinal data associations, associations with different SNPs and cognitive performance have been found, as in studies with a cumulative score, combining more than one allele (16, 38, 39).

For our three alleles, few studies are found related to cognitive performance; To *MOBP* rs1768208^T^ allele, Massimo and col. found that this allele is associated with a disruption of white matter networks in frontal regions, whereby *MOBP* rs1768208^T^ + individuals demonstrated faster rates of decline in executive function through time (16). Moreover, *MOBP* rs1768208 has been independently identified as a risk factor in confirmed cases of corticobasal degeneration (DCB) and in cases of PSP (40). Literature on the *APOE* rs429358 and cognitive processes yields variable results: some studies conducted in healthy adults have found associations with deficits in naming and orientation skills (41), while others have described better cognitive performances measured by the Mini-Mental State Examination (MMSE) (42). Specifically for APOE rs429358^C^, there are no association studies with neurocognitive tests, but Xue-Bin Li et al. suggest that the APOE rs429358^C^ allele genotype is associated with an increased risk of developing post-stroke depression, and may be detrimental to the recovery of nerve function after stroke (43). For *STX6*, there are no studies of its association with cognitive performance in this SNP to date. However, Ferrari and col. demonstrated that the rs1411478^A^ allele has a significantly lower expression of STX6 in white matter but not in any other brain region in PSP (23). As mentioned previously, *MOBP* rs1768208^T^ and *STX6* rs1411478^A^ alleles have been associated with disrupting white matter. This has revealed that cognitive performances are related to cortical thickness in frontotemporal regions and degradation in white matter integrity (35, 44, 45).

In conclusion, as no preliminary studies have been performed regarding the associations between cognitive performance and these SNPs in FTD, these results highlight the value of incorporating multiple biomarkers to help disentangle the mechanistic heterogeneity of cognitive decline (46). Our results may constitute a starting point for future studies involving these SNPs and their relationship with cognitive performance in patients with a probable diagnosis of FTD.

## Data availability statement

The original contributions presented in the study are included in the article. Requests to access the datasets should be directed to http://hdl.handle.net/10554/61347 or to the corresponding authors.

## Ethics statement

The studies involving human participants were reviewed and approved by Ethics Committee of the Pontificia Universidad Javeriana in Bogotá, Colombia approved the study. The patients/participants provided their written informed consent to participate in this study.

## Author contributions

AL-C, FC-S, PM, and PA-R developed the study concept and the study design. AL-C, FC-S, and PM performed testing and data collection. AL-C, FC-S, PM, AS, SG-N, and PA-R data analysis and interpretation under the supervision of IZ and DM. AL-C, FC-S, PM, AS, SG-N, PA-R, IZ, and DM drafted the manuscript. DM and IZ provided critical revisions. All authors contributed to the article and approved the submitted version.

## Funding

US-South American Initiative for Genetic-Neural-Behavioral Interactions in Human Neurodegenerative Research (R01AG057234-01A1), The Institute of Memory and Cognition (INTELLECTUS) the National Program of Science, Technology and Innovation in Health APS. This work was supported by, Colciencias 371-2011, 370-201 code: 120354531693.

## Conflict of interest

The authors declare that the research was conducted in the absence of any commercial or financial relationships that could be construed as a potential conflict of interest.

## Publisher's note

All claims expressed in this article are solely those of the authors and do not necessarily represent those of their affiliated organizations, or those of the publisher, the editors and the reviewers. Any product that may be evaluated in this article, or claim that may be made by its manufacturer, is not guaranteed or endorsed by the publisher.
