# Supplementary material for: Association between risk polymorphisms for neurodegenerative diseases and cognition in colombian patients with frontotemporal dementia
Source: Front Neurol. 2022 Aug 22;13:675301. doi: 10.3389/fneur.2022.675301 (PMC9443520; doi:10.3389/fneur.2022.675301)
Supplement: Supplementary file 1 [file Table_1.DOCX]

**Supplementary Material**

**Annex 1. SNP content in the Sequenom MassARRAY platform evaluated in genes associated with neurodegenerative diseases**

| **SNP ID** | **Gene** | **SNP ID** | **Gene** | **SNP ID** | **Gene** |
| --- | --- | --- | --- | --- | --- |
| rs2814707 | *C9orf72* | rs4147929 | *ABCA7* | rs7571971 | *EIF2AK3* |
| rs3849942 | *C9orf72* | rs3752246 | *ABCA7* | rs4499362 | *EPC2* |
| rs5848 | *GRN* | rs429358 | *APOE* | rs10808026 | *EPHA1* |
| rs12546767 | *WASHC5* | rs7412 | *APOE* | rs11767557 | *EPHA1* |
| rs1052553 | *MAPT* | rs4663105 | *BIN1* | rs2493013 | *EXOC2* |
| rs8070723 | *MAPT* | rs6733839 | *BIN1* | rs6852535 | *IL2/IL21* |
| rs242557 | *MAPT* | rs2142991 | *BMS1* | rs12203592 | *IRF4* |
| rs1020004 | *TMEM106* | rs10948363 | *CD2AP* | rs1491942 | *LRRK2* |
| rs1990622 | *TMEM106* | rs9349407 | *CD2AP* | rs1768208 | *MOBP* |
| RS1468803 | *TMEM106* | rs3865444 | *CD33* | rs4938933 | *MS4A6A* |
| rs3173615 | *TMEM106* | rs6547705 | *CD8B* | rs6687758 | *None/PSP* |
| rs3807865 | *TMEM106* | rs11136000 | *CLU* | rs3851179 | *PICALM* |
| rs12608932 | *UNC13A* | rs6656401 | *CR1* | rs561655 | *PICALM* |
| rs646776 | *CELSR2* | rs6701713 | *CR1* | rs17398575 | *PIK3CG* |
| rs11218343 | *SORL1* | rs28834970 | *PTK2B* | rs11568563 | *SLCO1A2* |
| rs1411478 | *STX6* | rs11781551 | *ZHX2* |  |  |

**Annex 2. Allele frequencies of the SNPs associated with FTD, and two SNPs associated with Alzheimer’s Disease in our cohort of FTD patients and their respective allele frequencies reported in population databases (1000 Genomes)**

| **Gene** | **SNP** | **Chro**  **mo-**  **some** | **Minor**  **allele** | **MAF** | **MAF** | **MAF** | **X2** | **p-value** | **X2** | **p-value** |
| --- | --- | --- | --- | --- | --- | --- | --- | --- | --- | --- |
|  |  |  |  | **Our cohort** | **1000**  **Genomes Global** | **1000 Genomes Colombia** | **Our Cohort vs 1000 Genomes Global** | **Our cohort vs 1000 Genomes Global** | **Our cohort vs 1000 Genomes Colombia** | **Our cohort vs 1000 Genomes Colombia** |
| ***MAPT*** | rs242557 | 17 | A | 0.3173 | 0.4167 | 0.3404 | 42.276 | 0.0399 | 0.2472 | 0.6191 |
| ***MAPT*** | rs1052553 | 17 | G | 0.1394 | 0.1177 | 0.1809 | 0.4716 | 0.4923 | 12.088 | 0.2716 |
| ***MAPT*** | rs8070723 | 17 | G | 0.1442 | 0.1456 | 0.1862 | 0.0016 | 0.9677 | 12.107 | 0.2712 |
| ***GRN*** | rs5848 | 17 | T | 0.2403 | 0.3864 | 0.3138 | 93.629 | 0.0022 | 26.092 | 0.1062 |
| ***C9orf72*** | rs2814707 | 9 | T | 0.1538 | 0.1749 | 0.3298 | 0.3208 | 0.5711 | 145.748 | 0.0001 |
| ***C9orf72*** | rs3849942 | 9 | T | 0.1942 | 0.1909 | 0.2181 | 0.0073 | 0.9318 | 0.3484 | 0.5550 |
| ***TMEM106*** | rs1020004 | 7 | C | 0.2211 | 0.2908 | 0.4574 | 24.498 | 0.1175 | 233.983 | < 0.001 |
| ***TMEM106*** | rs1990622 | 7 | A | 0.524 | 0.4391 | 0.5053 | 30.437 | 0.0811 | 0.1455 | 0.7029 |
| ***TMEM106*** | rs1468803 | 7 | A | 0.5336 | 0.4409 | 0.7447 | 36.255 | 0.0569 | 243.769 | < 0.001 |
| ***TMEM106*** | rs3173615 | 7 | C | 0.4759 | 0.4515 | 0.5053 | 0.2500 | 0.6171 | 0.3596 | 0.5487 |
| ***TMEM106*** | rs3807865 | 7 | G | 0.5528 | 0.4739 | 0.5213 | 25.968 | 0.1071 | 0.4135 | 0.5202 |
| ***KIAA0196*** | **rs12546767** | **8** | **C** | **0.375** | **0.1561** | **0.1596** | **378.295** | **0.0000** | **359.754** | **< 0.001** |
| ***UNC13A*** | rs12608932 | 19 | C | 0.4038 | 0.4322 | 0.3191 | 0.3418 | 0.5588 | 34.339 | 0.0639 |
| ***CELSR1*** | rs646776 | 1 | C | 0.2596 | 0.212 | 0.234 | 14.105 | 0.2350 | 0.3802 | 0.5375 |

MAF: minor allele frequency
